# Physical impairments among adults in Denmark: a register-based study

**DOI:** 10.1186/s12889-022-14747-9

**Published:** 2022-12-23

**Authors:** Helene Nikolajsen, Camilla Marie Larsen, Anders Holsgaard-Larsen, Birgit Juul-Kristensen, Lise Hestbaek

**Affiliations:** 1 Department of Sports Science and Clinical Biomechanics, University of Southern Denmark, Odense, Denmark; 2 Department of Physiotherapy, Institute of Health Studies, University College South Denmark, Esbjerg-Haderslev, Denmark; 3 Health Sciences Research Centre, UCL University College, Odense, Denmark; 4 Orthopaedic Research Unit, Department of Clinical Research, University of Southern Denmark, Odense, Denmark; 5 Department of Orthopaedics and Traumatology, Odense University Hospital, Odense, Denmark

**Keywords:** Physical impairment, Prevalence, Demography, Socio-demographic factors, Socio-economic factors, Population characteristics, Registry

## Abstract

**Background:**

Knowledge of the prevalence and socio-demographic characteristics of physical impairments is limited. This study aimed to determine the prevalence of physical impairments among adults in Denmark, both in total and according to nine common diagnostic subgroups, describe the socio-demographic and socio-economic profile, and compare the data with those of the general adult population.

**Methods:**

This descriptive, cross-sectional, register-based study evaluated the general socio-demographic and socio-economic variables, including sex, age, geographical region, origin, educational level, occupation, marital status, and disability level, of adults with physical impairments extracted by diagnosis from the Danish National Patient Register and Statistics Denmark by 31 December 2018. These data were compared with those of the general adult population in Denmark extracted from Statistics Denmark by January 2019.

**Results:**

In total, 606,857 adults with physical impairments were identified. Of the nine selected diagnoses, osteoarthritis (69.4%) was the most prevalent, followed by acquired brain injury (29.0%), rheumatoid arthritis (6.7%), multiple sclerosis (2.6%), spinal cord injury (1.5%), cerebral palsy (1.2%), amputation (0.7%), muscular dystrophy (0.5%), and poliomyelitis (< 0.1%). There were large variations in the socio-demographic and socio-economic profile between the nine diagnostic subgroups. The adults with physical impairments were more often women, were older, were less often immigrants and employed adults, had a lower educational level, and were more commonly married than the general adult population. Only the geographical region did not differ.

**Conclusion:**

The nine subgroups with diagnoses related to the musculoskeletal system represent 13% of the adult Danish population. The socio-demographic and socio-economic profile varied largely between the nine diagnostic subgroups, and almost all variables differed significantly between adults with physical impairments and the general adult population in Denmark. These findings reveal patterns and trends on socio-demographic and socio-economic variables essential for future planning at a societal level, including the healthcare and social sectors.

**Supplementary Information:**

The online version contains supplementary material available at 10.1186/s12889-022-14747-9.

## Background

People with physical impairments are a vulnerable group. as they have daily individual restrictions due to their impairment depending on the disability level and they are twice as likely to be physically inactive than people without impairments [1, 2]. Accordingly, these patients have an increased risk for both lifestyle-related morbidities and mortalities, leading to an individual and a societal economic burden [1, 3, 4]. Further, they more frequently develop other chronic diseases and conditions earlier than people without physical impairments [5]. Compared with the general population, people with physical impairments in Denmark lag behind in 9 of 10 indicators of the Disability Index: (1) equality and non-discrimination, (2) violence, (3) accessibility and mobility, (4) freedom and personal integrity, (5) living independently and being included in the community, (6) education, (7) health, (8) employment, and (9) social protection (only participation in political life was not affected). This trend appears to continue based on recent data [6]. As people with physical impairments currently live longer than they did previously, they constitute a growing group, accounting for approximately 1 billion people worldwide or about 15% of the world’s population in 2010 [7].

The inclusion of people with physical impairments in physical and sports activities, which has been shown to improve health and prevent lifestyle-related diseases in both adults with and without impairments [8], requires an infrastructure matching the needs of both groups. The inclusion in social and cultural activities is also essential to allow people with physical impairments to enjoy these activities with healthy relatives and friends on equal terms. However, when people with physical impairments attempt to engage in such activities, they encounter several barriers, including inaccessible environments or intra- or interpersonal issues [2, 9, 10]. This may be attributed to the limited knowledge about people with impairments and their experiences with such activities.

There is a need for more specific knowledge on the similarities and dissimilarities of the characteristics of people with and without impairments to facilitate opportunities for such activities. To date, there are limited valid data on the prevalence and characteristics of specific impairments, as the present knowledge is based on small groups not always representative of the broader population of adults with physical impairments [11–14] or on single diagnoses (e.g. multiple sclerosis [15] or cerebral palsy [16]) often without socio-demographic information [17]. The combination of data on the prevalence and socio-demographic characteristics can provide a more detailed overview of people with physical impairments, which can be useful within various fields (e.g. healthcare, social, and cultural sectors or socio-economic estimation or budgeting). The World Health Organization (WHO) also encourages disaggregation of the data on the prevalence of physical impairments into socio-demographic variables, including sex, age, income, and occupation, to identify patterns, trends, and other information about people with impairments [18].

For comparison across countries, International Classification of Diseases 10th revision (ICD-10) diagnostic codes have been used, although they address ‘impairments’ rather than ‘disabilities’. Accordingly, Denmark is an optimum study setting owing to the detailed registration of Danish citizens in several public registries including detailed information on patient diagnoses from all hospital contacts as well as a broad spectrum of socio-demographic data.

Therefore, the objectives of the current study were as follows: (1) determine the prevalence of nine selected diagnoses associated with physical impairments among adults in Denmark identified through a nationwide hospital register (i.e. osteoarthritis, acquired brain injury, rheumatoid arthritis, multiple sclerosis, spinal cord injury, cerebral palsy, amputation, muscular dystrophy, and poliomyelitis); (2) describe the socio-demographic and socio-economic profiles (i.e. sex, age, geographical region, origin, educational level, occupation, marital status, and disability level) of the total group of adults with physical impairments and each of the nine subgroups; and (3) compare the socio-demographic profile between the adults with physical impairments and the general adult population in Denmark.

## Methods

### Design

This descriptive, cross-sectional, register-based study used data available by 31 December 2018 from the Danish National Patient Register (DNPR) and Statistics Denmark. The STROBE guidelines [19] were used for study reporting.

### Population

The study population consisted of adults with physical impairments identified from the DNPR. Physical impairments included the following nine diagnoses with physical symptoms primarily related to the musculoskeletal system causing mobility problems: (1) osteoarthritis, (2) acquired brain injury, (3) rheumatoid arthritis, (4) multiple sclerosis, (5) spinal cord injury, (6) cerebral palsy, (7) amputation, (8) muscular dystrophy, and (9) poliomyelitis.

The dataset was created by Statistics Denmark, employing the following inclusion criteria: one or more ICD-10 diagnostic codes related to the nine diagnoses (see Appendix 1 for further details) and hospital admission from 1994 onwards. The ICD-10 diagnostic codes were extracted from the DNPR and included both A (primary diagnosis) and B diagnoses (optional secondary diagnosis during hospital admission). Both A and B diagnoses were used to identify the current population. The participants with physical impairments also had to be at least 18 years of age, alive, and living in Denmark by 31 December 2018.

### Data acquisition

The DNPR [20] was used to identify the population with physical impairments. It contains information about all diagnoses and performed operations since 1977 at all Danish Hospitals. Reporting to this national register is mandatory for all public and private hospitals, ensuring a valid and representative register covering all hospital admissions in Denmark. This government-funded registry was established by the National Board of Health, which provides an updated copy of the register to Statistics Denmark to allow research linkage to other registries [21].

Statistics Denmark is a governmental institution that collects and maintains electronic records for a broad spectrum of statistical and scientific purposes and has a large data quantity at its disposal [21]. In addition to the DNPR, we obtained data from the following registers: Population in Denmark, Educational Attainment, Danish Employment Classification Module, and Disability/Handicap Services.

All data sources were linked using the civil personal registration number, a unique identifier assigned to all Danish residents since 1968 that encodes their sex and date of birth. Accordingly, it was possible to link data from one or more registers or from other sources at an individual level. All linkage was performed within Statistics Denmark.

To compare the adults with physical impairments with the general adult population in Denmark, we used StatBank Denmark (www.statbank.dk), hosted by Statistics Denmark. This database is directly accessible and free of charge, and data are presented at an aggregate level to ensure non-identification of individuals and companies. All variables were categorised in the same manner as that in our population with physical impairments. We extracted data from all people who were aged 18 years or above, alive, and living in Denmark by 1 January 2019, except for data on the educational level, which were extracted from those aged 15–69 years only (*n* = 4,029,097).

### Variables

The following socio-demographic variables of the population with physical impairments were extracted from the four different registers in Statistics Denmark:

#### Population in Denmark

- Sex (binominal data).
- Age (ratio interval data) extracted by 31 December 2018 and grouped into ‘18–24’, ‘25–34’, ‘35–44’, ‘45–54’, ‘55–64’, ‘65–74’, and ’75 or above’ years.
- Geographical region in Denmark (nominal data) based on the participants’ home address by 31 December 2018 and categorised into ‘North Denmark’, ‘Central Denmark’, ‘Southern Denmark’, ‘Capital’, and ‘Zealand’.
- Marital status (nominal data) extracted and categorised into ‘unmarried’, ‘married or separated’, ‘divorced’, and ‘widow or widower’.
- Origin (nominal data) categorised into ‘Danish’, ‘immigrants’, or ‘descendants of immigrants’.

#### Educational Attainment

- Educational level (ordinal data) operationalised as the highest completed education and categorised into five groups according to the International Standard Classification of Education (ISCED) [22]: ‘ISCED 0–2’: primary and lower secondary school, ‘ISCED 3–4’: upper secondary school/vocational education, ‘ISCED 5–6’: bachelor or equivalent level, ‘ISCED 7–8’: master/doctoral level, and ‘unknown or missing’.

#### Employment classification Module

- Occupational status (nominal data) extracted and categorised into ‘affiliation to the labour market’, ‘education’, ‘unemployment or welfare payment’, ‘early retirement’, ‘retirement’, and ‘unknown or missing’.

#### Disability/Handicap services

- Functional level (ordinal) registered by municipalities as the overall functional status of a person who receives disability services and reported as follows: ‘no difficulties’, ‘slight difficulties’, ‘moderate difficulties’, ‘severe difficulties’, and ‘extreme difficulties’.

### Analysis

All analyses were descriptive, and statistical tests were performed only for comparison with the entire Danish population. The prevalence of physical impairments in the nine diagnostic groups, combined and by group, was reported as proportions of adult citizens living in Denmark by 31 December 2018. The distribution of sex, age, geographical region, origin, educational level, occupation, and marital status within the nine diagnostic subgroups and the entire study group was estimated as proportions with 95% confidence intervals. Further, all variables of the population with physical impairments were compared with those of the general Danish population. To ensure data protection, we did not report the data when there were fewer than 10 individuals in a cell.

Pearson’s chi-squared test of independence was used to calculate *p* values for differences in the distribution of the variables between the diagnostic subgroups and the general Danish population. We excluded the diagnostic subgroups from the total Danish population before calculating the *p* values (see Appendix 2 for additional information on the outcome of the chi-squared test).

Missing or unknown data were excluded from the analysis. The significance level was set at *p* < 0.05, and all analyses were performed using STATA 16.1 [23]. Finally, the distribution of the functional level of the nine diagnostic subgroups and the total group with physical impairments was reported.

### Ethics

The project was approved by the Research & Innovation Organisation, University of Southern Denmark on behalf of the Danish Data Protection Agency (number 2015-57-0008).

## Results

### Prevalence

**Fig. 1:**
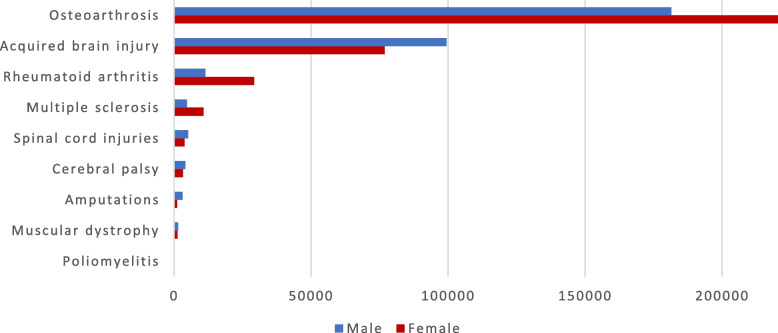
Nine diagnostic subgroups^1^ including a total of 606,857 adults. Data were extracted from the Danish National Patient Register. ^1^Proportion of adults who were alive and living in Denmark by 31 December 2018, determined to have one of the diagnoses listed in Appendix 1 between January 1994 and December 2018

In total, 606,857 patients were included in the nine diagnostic subgroups, equivalent to 13% of the total adult population in Denmark. The largest diagnostic subgroup was the subgroup with osteoarthritis (67.4%), followed by those with acquired brain injury (29.0%), rheumatoid arthritis (6.7%), multiple sclerosis (2.6%), spinal cord injury (1.5%), cerebral palsy (1.2%), amputation (0.7%), muscular dystrophy (0.5%), and poliomyelitis (< 0.1%) (Fig. 1). Almost 91% were included in only one subgroup, approximately 9% in two subgroups, and < 0.5% in three or more subgroups.

### Socio-demographic variables

The socio-demographic variables, including sex, age, geographical region, and origin, of all subgroups, except for the subgroup with poliomyelitis since several cells included fewer than 10 individuals, are shown in Table 1. The comparison of the socio-demographic variables between the total group with physical impairments and the general adult population is shown in Table 3.

**Table 1:** Distribution of demographic variables in eight diagnostic subgroups^a^

	Osteoarthritis *n* = 409,202	Acquired brain injury *n* = 176,276	Rheumatoid arthritis *n* = 40,590	Multiple sclerosis *n* = 15,496	Spinal cord injury *n* = 8,922	Cerebral palsy *n* = 7,336	Amputation *n* = 4,191	Muscular dystrophy *n* = 2,751
**Sex**								
Male	**44.34 (44.18–44.49)**	**56.41 (56.18–56.64)**	**28.11 (27.67–28.55)**	**30.60 (29.88–31.33)**	**57.61 (56.58–58.64)**	**55.49 (54.35–56.64)**	**72.87 (71.50–74.21)**	**53.22 (51.33–55.10)**
Female	**55.66 (55.51–55.82)**	**43.59 (43.36–43.82)**	**71.89 (71.45–72.33)**	**69.40 (68.67–70.12)**	**42.39 (41.36–43.42)**	**44.51 (43.36–45.65)**	**27.13 (25.79–28.50)**	**46.78 (44.90–48.67)**
**Age (y)**								
Men								
18–24	**0.12 (0.11–0.13)**	**1.37 (1.31–1.42)**	**0.30 (0.25–0.36)**	**0.37 (0.28–0.48)**	**6.18 (5.69–6.70)**	**15.42 (14.60–16.26)**	**2.60 (2.14–3.13)**	**7.42 (6.46–8.46)**
25–34	**0.70 (0.68–0.73)**	**2.96 (2.88–3.04)**	**0.93 (0.84–1.02)**	**2.17 (1.94–2.41)**	**8.48 (7.91–9.08)**	**16.93 (16.08–17.81)**	**7.95 (7.14–8.81)**	**10.83 (9.70–12.05)**
35–44	**1.98 (1.94–2.02)**	**3.43 (3.35–3.52)**	**1.67 (1.54–1.79)**	**5.15 (4.81–5.51)**	**8.17 (7.61–8.76)**	**8.41 (7.79–9.07)**	**11.36 (10.41–12.36)**	**8.29 (7.28–9.38)**
45–54	**5.89 (5.82–5.96)**	**5.74 (5.63–5.85)**	**3.76 (3.58–3.95)**	**7.79 (7.37–8.22)**	**10.35 (9.72–11.00)**	**5.32 (4.81–5.85)**	**16.56 (15.45–17.72)**	**9.85 (8.76–11.03)**
55–64	**10.05 (9.96–10.15)**	**9.68 (9.54–9.82)**	**5.85 (5.62–6.08)**	**7.69 (7.27–8.12)**	**10.14 (9.52–10.79)**	**4.85 (4.37–5.37)**	**15.92 (14.82–17.06)**	**8.18 (7.18–9.27)**
65–74	**13.49 (13.38–13.59)**	**15.44 (15.27–15.61)**	**8.08 (7.82–8.35)**	**5.52 (5.16–5.89)**	**9.10 (8.51–9.72)**	**3.05 (2.67–3.47)**	**11.83 (10.87–12.85)**	**5.56 (4.73–6.48)**
≥ 75	**12.10 (12.00–12.20)**	**17.80 (17.62–17.98)**	**7.53 (7.28–7.79)**	**1.92 (1.71–2.15)**	**5.19 (4.74–5.67)**	**1.51 (1.25–1.82)**	**6.66 (5.92–7.45)**	**3.09 (2.48–3.81)**
Women								
18–24	**0.14 (0.13–0.16)**	**0.87 (0.82–0.91)**	**0.66 (0.58–0.74)**	**0.89 (0.75–1.05)**	4.64 (4.21–5.10)	**11.14 (10.43–11.88)**	**1.53 (1.18–1.95)**	**4.47 (3.73–5.31)**
25–34	**0.56 (0.53–0.58)**	**1.50 (1.44–1.55)**	**2.63 (2.48–2.79)**	**5.38 (5.03–5.75)**	6.46 (5.95–6.99)	**12.38 (11.63–13.15)**	**3.05 (2.55–3.62)**	**6.91 (5.99–7.92)**
35–44	**1.71 (1.67–1.75)**	**2.03 (1.97–2.10)**	**5.34 (5.12–5.56)**	**12.03 (11.52–12.55)**	6.09 (5.60–6.60)	**6.64 (6.08–7.23)**	**3.82 (3.26–4.44)**	**7.78 (6.81–8.84)**
45–54	**5.82 (5.74–5.89)**	**3.86 (3.77–3.95)**	**9.87 (9.58–10.16)**	**18.17 (17.56–18.78)**	6.89 (6.38–7.44)	**5.23 (4.74–5.77)**	**5.01 (4.37–5.72)**	**9.63 (8.56–10.80)**
55–64	**11.12 (11.02–11.21)**	**6.36 (6.25–6.48)**	**14.78 (14.44–15.13)**	**17.14 (16.55–17.74)**	6.90 (6.39–7.45)	**4.80 (4.32–5.31)**	**6.23 (5.51–7.00)**	**8.76 (7.73–9.88)**
65–74	**16.42 (16.31–16.53)**	**10.25 (10.11–10.39)**	**19.04 (18.66–19.43)**	**11.53 (11.03–12.05)**	6.39 (5.89–6.92)	**1.61 (1.33–1.92)**	**4.37 (3.77–5.03)**	**5.78 (4.94–6.72)**
≥ 75	**19.91 (19.78–20.03)**	**18.72 (18.54–18.90)**	**19.58 (19.19–19.97)**	**4.26 (3.95–4.59)**	5.02 (4.58–5.49)	**1.76 (1.47–2.09)**	**3.13 (2.62–3.70)**	**3.45 (2.80–4.21)**
**Geographical region**								
North Denmark	**10.82 (10.72–10.91)**	**10.02 (9.88–10.16)**	**9.46 (9.18–9.75)**	**8.98 (8.54–9.44)**	**9.33 (8.73–9.95)**	**9.99 (9.31–10.70)**	**12.79 (11.79–13.84)**	9.85 (8.76–11.03)
Central Denmark	**23.52 (23.39–23.65)**	**20.84 (20.66–21.03)**	**19.45 (19.07–19.84)**	**22.01 (21.36–22.67)**	**23.26 (22.38–24.15)**	**19.49 (18.59–20.42)**	**20.11 (18.91–21.36)**	22.68 (21.13–24.29)
Southern Denmark	**23.61 (23.48–23.74)**	**22.86 (22.66–23.06)**	**21.62 (21.22–22.02)**	**22.43 (21.78–23.10)**	**20.62 (19.79–21.48)**	**23.34 (22.37–24.32)**	**31.23 (29.83–32.66)**	20.61 (19.11–22.17)
Capital	**26.72 (26.58–26.86)**	**29.43 (29.22–29.65)**	**30.34 (29.89–30.79)**	**30.74 (30.02–31.48)**	**30.23 (29.28–31.19)**	**29.88 (28.83–30.94)**	**20.50 (19.28–21.75)**	31.08 (29.35–32.85)
Zealand	**15.33 (15.22–15.44)**	**16.84 (16.67–17.02)**	**19.14 (18.75–19.52)**	**15.83 (15.26–16.41)**	**16.57 (15.80–17.35)**	**17.30 (16.44–18.18)**	**15.37 (14.29–16.49)**	15.78 (14.43–17.19)
**Origin**								
Danish	**93.22 (93.14–93.29)**	**93.84 (93.72–93.95)**	**92.99 (92.74–93.24)**	**94.10 (93.72–94.47)**	**91.00 (90.39–91.59)**	**91.56 (90.90–92.19)**	**90.19 (89.25–91.08)**	**92.44 (91.39–93.40)**
Immigrants	**6.52 (6.44–6.60)**	**5.59 (5.48–5.70)**	**6.57 (6.33–6.81)**	**4.82 (4.49–5.17)**	**7.08 (6.56–7.64)**	**4.65 (4.18–5.16)**	**8.88 (8.03–9.78)**	**5.63 (4.80–6.56)**
Descendant of immigrants	**0.26 (0.25–0.28)**	**0.57 (0.54–0.61)**	**0.44 (0.38–0.51)**	**1.08 (0.92–1.25)**	**1.92 (1.64–2.22)**	**3.79 (3.36–4.25)**	**0.93 (0.66–1.27)**	**1.93 (1.45–2.51)**

Data are reported as proportions^b^ with 95% confidence intervals

Distributions written in bold were significantly different from the distribution of the general Danish population^b^ (*p* < 0.001) (each diagnostic subgroup is excluded from the general adult population before comparison)

^a^Poliomyelitis is not included owing to a small number of cases

^b^General adult population alive and living in Denmark by 1 January 2019

There were more women in the total group with physical impairments than in the entire Danish population (Table 3), but there were also large sex differences between the diagnostic subgroups (Table 1). Rheumatoid arthritis and multiple sclerosis were considerably more frequent among the women than among the men (72% vs. 28% and 69% vs. 31%, respectively). In contrast, injury-related impairments were more common among the men than among the women, with 73% of those with amputation and 58% of those with spinal cord injury being men. Acquired brain injuries, which could be related to trauma in some cases, were more frequent among the men than among the women (men: 56% vs. women: 44%). There were only minor sex differences between the subgroups with osteoarthritis, cerebral palsy, muscular dystrophy, and poliomyelitis. The sex difference between the total group with physical impairments and the general adult population was significant for all diagnoses (*p* ≤ 0.001) (Appendix 2a).

Age differed between the nine subgroups, with the subgroup with cerebral palsy having the largest proportion of young people and the subgroup with acquired brain injury having the oldest people (Table 1).

The proportion of both men and women aged above 75 years was higher in the total group with physical impairments than in the general adult population (Table 3). This difference was attributed to the proportion in the two largest subgroups – osteoarthritis and acquired brain injury, including apoplexia, which both usually appear later in life. The age distribution between the diagnostic subgroups according to sex and the general adult population significantly differed (*p* ≤ 0.001), except for the women with spinal cord injury (*p* = 0.341) (Table 1 and Appendix 2b and c).

The distribution of the selected diagnostic subgroups across the Danish geographical regions generally followed the same pattern as that of the general adult population, except for amputations, which were much more prevalent in Southern Denmark and less prevalent in the Capital region. Rheumatoid arthritis was more prevalent in the Zealand region, while osteoarthritis was less prevalent in the Capital region. The geographical distribution between all diagnostic subgroups and the general adult population significantly differed (*p* ≤ 0.001), except for the subgroup with muscular dystrophy (*p* = 0.367) (Table 1 and Appendix 2d).

The immigrants both from western and non-western countries as well as the descendants of immigrants were only half more likely to have a physical impairment than the general adult population (Table 3). However, cerebral palsy was twice more common in the descendants of immigrants than in the general adult population (3.8% vs. 1.7%). The distribution of origin between all diagnostic subgroups and the general adult population significantly differed (*p* ≤ 0.001) (Table 1 and Appendix 2e).

### Socio-economic variables

The socio-economic variables, including the educational level, occupation, and marital status, of all subgroups, except for the subgroup with poliomyelitis owing to a small number of cases, are shown in Table 2. The comparison of the socio-economic variables between the total group with physical impairments and the general adult population is shown in Table 3.

**Table 2:** Distribution of socio-economic variables in eight diagnostic subgroups^a^

	Osteoarthritis *n* = 409,202	Acquired brain injury *n* = 176,276	Rheumatoid arthritis *n* = 40,590	Multiple sclerosis *n* = 15,496	Spinal cord injury *n* = 8,922	Cerebral palsy *n* = 7,336	Amputation *n* = 4,191	Muscular dystrophy *n* = 2,751
**Educational level**^**c,d**^								
ISCED 0–2	**33.00 (32.86–33.14)**	**36.67 (36.45–36.90)**	**33.88 (33.42–34.35)**	**23.43 (22.77–24.11)**	**45.19 (44.16–46.23)**	**66.25 (65.15–67.33)**	**31.19 (29.78–32.61)**	**35.66 (33.87–37.48)**
ISCED 3–4	**41.84 (41.69–41.99)**	**40.60 (40.37–40.83)**	**39.60 (39.12–40.07)**	**43.13 (42.35–43.92)**	**31.76 (30.80–32.74)**	**18.50 (17.62–19.41)**	**47.79 (46.27–49.32)**	**37.88 (36.06–39.72)**
ISCED 5–6	**18.74 (18.62–18.86)**	**15.37 (15.20–15.54)**	**19.59 (19.20–19.98)**	**23.60 (22.93–24.28)**	**14.46 (13.73–15.21)**	**7.73 (7.13–8.36)**	**14.75 (13.69–15.86)**	**17.70 (16.29–19.18)**
ISCED 7–8	**4.58 (4.52–4.65)**	**5.12 (5.02–5.22)**	**5.38 (5.17–5.61)**	**9.01 (8.56–9.47)**	**5.74 (5.26–6.24)**	**3.37 (2.97–3.81)**	**4.51 (3.90–5.18)**	**7.49 (6.53–8.54)**
Unknown or missing	**1.84 (1.80–1.88)**	**2.24 (2.18–2.31)**	**1.55 (1.43–1.67)**	**0.83 (0.69–0.98)**	**2.85 (2.51–3.21)**	**4.16 (3.71–4.64)**	**1.77 (1.39–2.21)**	**1.27 (0.89–1.77)**
**Occupation**								
Affiliation to the labour market	**28.76 (28.62–28.90)**	**21.79 (21.60–21.98)**	**28.32 (27.88–28.76)**	**35.53 (34.78–36.29)**	**18.49 (17.69–19.32)**	**16.43 (15.58–17.29)**	**48.51 (46.99–50.03)**	**30.21 (28.50–31.96)**
Education	**0.31 (0.30–0.33)**	**1.53 (1.48–1.59)**	**1.07 (0.97–1.17)**	**1.34 (1.17–1.54)**	**4.60 (4.17–5.05)**	**10.37 (9.68–11.09)**	**3.15 (2.64–3.72)**	**7.34 (6.40–8.38)**
Unemployment or welfare payment	**5.00 (4.93–5.06)**	**5.57 (5.46–5.67)**	**6.74 (6.50–6.99)**	**11.08 (10.59–11.59)**	**8.61 (8.03–9.21)**	**8.49 (7.86–9.15)**	**9.59 (8.72–10.52)**	**11.27 (10.11–12.51)**
Early retirement	**7.80 (7.72–7.89)**	**11.10 (10.95–11.25)**	**11.71 (11.40–12.03)**	**29.19 (28.47–29.91)**	**42.52 (41.50–43.56)**	**53.87 (52.72–55.02)**	**13.34 (12.32–14.40)**	**32.93 (31.18–34.73)**
Retirement	**57.25 (57.10–57.40)**	**58.82 (58.59–59.05)**	**50.92 (50.44–51.41)**	**21.41 (20.77–22.07)**	**24.23 (23.35–25.14)**	**8.41 (7.79–9.07)**	**23.17 (21.90–24.48)**	**16.32 (14.96–17.76)**
Unknown or missing	**0.88 (0.85–0.91)**	**1.19 (1.14–1.24)**	**1.23 (1.12–1.34)**	**1.45 (1.26–1.65)**	**1.55 (1.30–1.82)**	**2.43 (2.09–2.80)**	**2.24 (1.82–2.74)**	**1.93 (1.45–2.51)**
**Marital status**								
Unmarried	**10.61 (10.52–10.71)**	**18.18 (18.00–18.36)**	**14.71 (14.36–15.06)**	**24.77 (24.09–25.46)**	**49.81 (48.77–50.85)**	**81.73 (80.83–82.61)**	**29.28 (27.90–30.68)**	**48.06 (46.17–49.94)**
Married or separated	**56.28 (56.13–56.43)**	**46.32 (46.09–46.56)**	**52.28 (51.79–52.77)**	**51.97 (51.18–52.76)**	**31.50 (30.53–32.47)**	**11.22 (10.51–11.96)**	**49.01 (47.49–50.54)**	**36.57 (34.77–38.40)**
Divorced	**16.07 (15.96–16.18)**	**17.21 (17.03–17.38)**	**16.74 (16.38–17.11)**	**17.61 (17.01–18.22)**	**13.19 (12.50–13.91)**	**5.10 (4.61–5.63)**	**16.37 (15.26–17.52)**	**11.60 (10.42–12.85)**
Widow or widower	**17.04 (16.92–17.15)**	**18.29 (18.11–18.47)**	**16.27 (15.91–16.64)**	**5.65 (5.29–6.03)**	**5.50 (5.04–6.00)**	**1.95 (1.65–2.29)**	**5.34 (4.68–6.07)**	**3.78 (3.10–4.56)**

Data are reported as proportions^b^ with 95% confidence intervals

Distributions written in bold were significantly different from the distribution of the general Danish population^b^ (*p* < 0.001) (each diagnostic subgroup is excluded from the general adult population before comparison)

^a^Poliomyelitis is not included owing to a small number of cases

^b^General adult population alive and living in Denmark by 1 January 2019

^c^Data on the educational level were obtained from adults aged 15–69 years (from StatDenmark; *n* = 4,029,097) and are therefore not directly comparable with those of the group with physical impairments

^d^ISCED (International Standard Classification of Education) 0–2: primary and lower secondary school, 3–4: upper secondary school/vocational education, 5–6: bachelor or equivalent level, 7–8: master/doctoral level

**Table 3:** Distribution of socio-demographic and socio-economic variables in the group with impairments^a^ and general adult population

	Total group with impairments^a^ *n* = 606,857	Total group with impairments^a^ 95% CI	General adult population in DK^b^ *n* = 4,645,697	General adult population in DK^b^ 95% CI
**Sex**				
Male	285,391	**47.03 (46.90–47.15)**	2,294,081	49.38 (49.34–49.43)
Female	321,466	**52.97 (52.85–53.10)**	2,351,616	50.62 (50.57–50.66)
**Age (y)**				
Men				
18–24	4,633	**0.76 (0.74–0.79)**	271,039	5.83 (5.81–5.86)
25–34	10,761	**1.77 (1.74–1.81)**	379,277	8.16 (8.14–8.19)
35–44	16,803	**2.77 (2.73–2.81)**	354,771	7.64 (7.61–7.66)
45–54	37,377	**6.16 (6.10–6.22)**	408,131	8.79 (8.76–8.81)
55–64	59,851	**9.86 (9.79–9.94)**	358,343	7.71 (7.69–7.74)
65–74	80,715	**13.30 (13.22–13.39)**	314,798	6.78 (6.75–6.80)
≥ 75	75,251	**12.40 (12.32–12.48)**	207,722	4.47 (4.45–4.49)
Women				
18–24	3,632	**0.60 (0.58–0.62)**	259,452	5.58 (5.56–5.61)
25–34	8,049	**1.33 (1.30–1.36)**	363,886	7.83 (7.81–7.86)
35–44	15,168	**2.50 (2.46–2.54)**	350,681	7.55 (7.52–7.57)
45–54	36,682	**6.04 (5.98–6.10)**	402,733	8.67 (8.64–8.69)
55–64	61,963	**10.21 (10.13–10.29)**	361,321	7.78 (7.75–7.80)
65–74	87,363	**14.40 (14.31–14.48)**	333,726	7.18 (7.16–7.21)
≥ 75	108,609	**17.90 (17.80–17.99)**	279,817	6.02 (6.00–6.04)
**Geographical region**				
North Denmark	64,222	**10.58 (10.51–10.66)**	476,109	10.25 (10.22–10.28)
Central Denmark	137,692	**22.69 (22.58–22.79)**	1,048,402	22.57 (22.53–22.61)
Southern Denmark	141,087	**23.25 (23.14–23.36)**	979,225	21.08 (21.04–21.12)
Capital	167,461	**27.59 (27.48–27.71)**	1,470,152	31.65 (31.60–31.69)
Zealand	96,395	**15.88 (15.79–15.98)**	671,809	14.46 (14.43–14.49)
**Origin**				
Danish	565,711	**93.22 (93.16–93.28)**	4,005,579	86.22 (86.19–86.25)
Immigrants	38,422	**6.33 (6.27–6.39)**	562,347	12.10 (12.08–12.13)
Descendant of immigrants	2,724	**0.45 (0.43–0.47)**	77,771	1.67 (1.66–1.69)
**Educational level**^**c,d**^				
ISCED 0–2	204,181	**33.65 (33.53–33.76)**	1,025,443	25.45 (25.41–25.49)
ISCED 3–4	250,753	**41.32 (41.20–41.44)**	1,606,866	39.88 (39.83–39.93)
ISCED 5–6	109,907	**18.11 (18.01–18.21)**	891,196	22.12 (22.08–22.16)
ISCED 7–8	30,418	**5.01 (4.96–5.07)**	435,718	10.81 (10.78–10.84)
Unknown or missing	11,598	**1.91 (1.88–1.95)**	69,874	1.73 (1.72–1.75)
**Occupation**				
Affiliation to the labour market	171,879	**28.32 (28.21–28.44)**	2,786,698	59.98 (59.94–60.03)
Education	5,881	**0.97 (0.94–0.99)**	202,840	4.37 (4.35–4.38)
Unemployment or welfare payment	34,424	**5.67 (5.61–5.73)**	278,150	5.99 (5.97–6.01)
Early retirement	61,365	**10.11 (10.04–10.19)**	223,007	4.80 (4.78–4.82)
Retirement	326,765	**53.85 (53.72–53.97)**	999,083	21.51 (21.47–21.54)
Unknown or missing	6,543	**1.08 (1.05–1.10)**	155,919	3.36 (3.34–3.37)
**Marital status**				
Unmarried	90,323	**14.88 (14.79–14.97)**	1,669,782	35.94 (35.90–35.99)
Married or separated	320,687	**52.84 (52.72–52.97)**	2,141,704	46.10 (46.06–46.15)
Divorced	98,226	**16.19 (16.09–16.28)**	545,085	11.73 (11.70–11.76)
Widow or widower	97,621	**16.09 (15.99–16.18)**	289,126	6.22 (6.20–6.25)

Data are reported as numbers and proportions with 95% confidence intervals^b^

Distributions written in bold were significantly different from the distribution of the general Danish population^b^ (*p* < 0.001) (the total group with physical impairments is excluded from the general adult population before comparison)

^a^Proportion of adults alive and living in Denmark by 31 December 2018, identified to have one of the diagnoses listed in Appendix 1 between January 1994 and December 2018

^b^General adult population alive and living in Denmark by 1 January 2019

^c^Data on the educational level were obtained from adults aged 15–69 years (from StatDenmark; *n* = 4,029,097) and are therefore not directly comparable with those of the group with physical impairments

^d^ISCED (International Standard Classification of Education) 0–2: primary and lower secondary school, 3–4: upper secondary school/vocational education, 5–6: bachelor or equivalent level, 7–8: master/doctoral level

The educational level was lower in all subgroups than in the general adult population (Table 3) but differed considerably between the subgroups (Table 2). Diagnoses with possible cognitive dysfunctions, including cerebral palsy and spinal cord injury, were associated with very low educational levels. The educational level of the adults with multiple sclerosis was similar to that of the general adult population. The distribution of the educational level significantly differed (*p* ≤ 0.001) between all subgroups (Table 2 and Appendix 2f).

Only about half as many people with impairments as the general adult population (60.0%) were affiliated to the labour market (28.3%) (Table 3). The subgroup with cerebral palsy had the lowest proportion of people affiliated to the labour market (16.4%), while the subgroup with amputation had the highest proportion of such (48.5%) (Table 2). The proportion of adults on early retirement (10% vs. 4.8%) or retirement (54.8% vs. 21.5%) was twice higher in the total group with physical impairments than in the general adult population (Table 3). The distribution of occupation significantly differed between all subgroups (*p* ≤ 0.001) and the general adult population (Table 2 and Appendix 2g).

The marital status also differed significantly between the groups, with only 14.9% of the adults with physical impairments being unmarried compared with 35.9% of the general adult population; accordingly, there were more people with physical impairments in the married/separated, divorced, and widow/widower groups (Table 3). There were large differences between the subgroups, with 18.3% of the patients with cerebral palsy and 89.4% of those with osteoarthritis being married. The distribution of the marital status between all subgroups and the general adult population significantly differed (*p* ≤ 0.001) (Table 2 and Appendix 2h).

### Disability level

The disability level was a new variable in the register, and only very few people were registered during the first year of reporting. The reporting rate was only 0.9% in the total group with physical impairments and differed considerably between the subgroups, ranging from 0.4% in the subgroup with osteoarthritis to 14.3% in the subgroup with cerebral palsy (Table 4).

**Table 4:** Disability level of eight diagnostic subgroups^a^ and the total group with physical impairments

	*n*	Reporting rate	Disability level				
			No difficulties	Slight difficulties	Moderate difficulties	Severe difficulties	Extreme difficulties
Osteoarthritis	1,444	0.4	1.0	20.0	49.3	25.4	4.2
Acquired brain injury	2,457	1.4	0.9	16.7	45.8	31.8	4.8
Rheumatoid arthritis	149	0.4	2.0	16.8	53.7	24.2	3.4
Multiple sclerosis	1,442	2.9	0.5	15.6	48.2	27.1	8.6
Spinal cord injury	797	8.9	0.4	4.8	24.8	45.2	24.8
Cerebral palsy	1,048	14.3	0.2	6.4	26.3	45.9	21.2
Amputation	44	1.0	2.3	22.7	34.1	38.6	2.3
Muscular dystrophy	152	5.5	0.7	3.3	38.2	46.7	11.2
Total group with physical impairments	5,412	0.9	0.8	14.9	42.4	33.5	8.5

Data are reported as proportions (%)

Due to common rounding rules, not all data add up to exactly 100% (Osteoarthritis 99.9%; Rheumatoid arthritis 100.1%; Muscular dystrophy 100.1%; Total group with physical impairment 100.1%)

^a^Adults alive and living in Denmark by 31 December 2018, identified to have one of the diagnoses listed in Appendix 1 between January 1994 and December 2018

Among the total group with physical impairments, 42% and 34% had moderate and severe difficulties, respectively. Five subgroups had about 16–25% of adults categorised in the two categories with the best functional levels: no difficulties and slight difficulties (amputation: 25%, osteoarthritis: 21%, rheumatoid arthritis: 18.8%, acquired brain injury: 17.6%, and multiple sclerosis: 16.1%). This was in contrast with the remaining three subgroups, wherein most adults were categorised into the two categories with the worst functional levels: severe difficulties and extreme difficulties (spinal cord injury: 70%, cerebral palsy: 67.1%, and muscular dystrophy: 57.8%).

## Discussion

To our knowledge, this is the first study to determine the prevalence of physical impairments among adults in Denmark, describe the socio-demographic and socio-economic variables from national register-based data, and compare these data with those of the general adult population in Denmark.

### Prevalence

In total, 606,857 adults were included in the nine diagnostic subgroups, equivalent to 13% of the total adult population in Denmark. Our present data are an example of a medical model, where physical impairment is strictly related to a somatic diagnosis and thus does not represent all impairments, including mental or sensory impairments. Although the existing literature describes the prevalence of disability, while the current study reports that of impairment, we still compared our data with existing findings, as we believe that this difference is partly attributed to inconsistencies of both concepts. The prevalence of physical impairments related to the musculoskeletal system (13%) is relatively high compared with the 20-year-old estimate from the WHO, reporting that about 15% of the world’s population aged > 15 years is living with some type of disability, but with the percentage including both mental and physical disabilities. However, an American survey based on six specific disability type questions estimated that 25% of non-institutionalised adults aged ≥ 18 years have some kind of disability [12]. Within this estimate, the most frequent condition with a prevalence of 13.7% was disability related to mobility (serious difficulty in walking or climbing stairs), equivalent to our estimate, which also focuses on impairment with mobility issues [12]. Studies using self-reported data may overestimate the prevalence compared with the present study using register-based data for diagnosis. One example is the Survey of Health, Impairment and Living Conditions in Denmark performed in 2012, 2016, and 2020 that investigated self-reported physical disabilities/long-lasting health conditions and reported a prevalence ranging from 24 to 27% among 16–64-year-old Danes [13, 24–27].

As previously described, the difference in the reported prevalence may be attributed to the different methods of measuring and defining disability [28]. From a health and welfare perspective, the most dominating models are ‘the medical model’ and ‘the social model’. Our data are solely based on the medical model, which focuses on disability as the diagnosis and impairment. In contrast, the social model distinguishes between impairment (related to the physical body and function) and disability (disadvantage or restriction of activity caused by the surrounding society).

### Socio-demographic variables

Our study showed that more women had impairments than men, consistent with previous Danish findings [29]. Autoimmune diseases such as rheumatoid arthritis and multiple sclerosis are highly predominant among women [17, 30], while diseases that can be traumatically induced such as amputations, acquired brain injuries, and spinal cord injuries are more common among men. Data from Danish hospital records show that men are more often involved in traffic accidents, work-related accidents, and violence than women [31].

The data on the age distribution reflect that the prevalence of osteoarthrosis and acquired brain injury (including stroke) increases with age, while the other diagnoses represent other pathological patterns with earlier disease onset and often earlier mortality. Furthermore, osteoarthritis was found to be less prevalent in the Capital region than in the other regions, probably reflecting a younger population in the Capital region [21.2% of the adult population was aged ≥ 65 years in the Capital region compared with 26.0% of the general adult population (data not shown)].

An interesting finding was the very high prevalence of cerebral palsy among the descendants of immigrants, consistent with the data of immigrants from Sweden [32] and Great Britain [33]. Consanguinity is suggested as a relevant factor influencing the prevalence of cerebral palsy, as high rates are reported among Turkish and Pakistani immigrants [33, 34], two of the largest immigrant groups in Denmark.

### Socio-economic variables

People with disabilities are known to have lower educational levels and less affiliations to the labour market [7]; this pattern is more apparent with an increased severity of the disability [26, 35] as well as with an early onset of the disability [36]. Our results also reflect this pattern but are unique, as they could be compared across diagnostic subgroups. The current data showed that the subgroups with early onset and/or cognitive dysfunction had lower educational levels, less affiliations with the labour market, and higher probabilities of disability pension or early retirement. In contrast, the subgroup with multiple sclerosis had an educational level almost similar to that of the general adult population in Denmark. This reflects that multiple sclerosis usually initially occurs around the age of 30 years [37, 38], wherein most people have completed their education and are working. This trend was also observed for osteoarthritis, which may to some degree be associated with the level of physical work load and is therefore more prevalent among blue collar workers [39].

More people were married or had been married in the total group than in the general adult population. The data on the marital status of people with impairments are limited. Nevertheless, the present results are in line with those of a previous study on Canadian women [40]. However, our findings do not provide information about whether people cohabit without being married.

### Disability level

Registration of the disability level by municipalities was introduced on 1 January 2018. Consequently, our data represent data from the first year of registration. Reporting of the disability level in this register was voluntary, as reflected by the very low reporting rates. Thus, data are very sparse, and the reliability and validity remain unknown. Nevertheless, they may provide a preliminary indication about the burden of the various diagnoses. The overall disability level was registered by social workers in the municipalities, intending to aid in the assessment of allocation of healthcare and social services. Such reporting is aspired to increase in the future, as it can describe the degree of disability across diagnoses.

### Strengths and limitations

The study strengths include the use of the DNPR, which collects data continuously through digitalised workflows and provides highly valid data of about 5.8 million people. Further, the linking of information using a personal identifier – the civil personal registration number – to demographic data stored by Statistics Denmark provides thoroughly complete and non-biased information [41], making it possible to conduct comparisons with the general adult Danish population. This ensured a large dataset and avoided attrition bias.

Another strength is that the present data are based on a medical model of measuring impairment, making them easily replicable; thus, similar studies may be performed internationally for comparison.

Meanwhile, the study limitations include the use of ICD-10 diagnostic codes, which date back only to 1994 in the DNPR; this indicates that we may have missed adults diagnosed before 1994. However, all patients admitted to the hospital between 1994 and 2018 with one of the selected ICD-10 diagnostic codes as a primary or secondary diagnosis were included in this study; therefore, we anticipate a very small number of missing cases. A potential source of overestimation is that individuals can be assigned a preliminary ICD-10 diagnostic code if they are under observation for a specific diagnosis. This will appear in registers and may represent false-positive diagnoses; cerebral palsy and spinal cord injury are especially at risk for this overrepresentation.

Information on the general adult population in Denmark has not been collected at an individual level but at an aggregated level from Statistics Denmark. Fortunately, we were able to collect information with a cut-off point that differed by only 1 day from the remaining data. However, a corresponding limitation is that the data on the educational level from the general adult population could only be limited to those aged 15–69 years and were therefore not directly comparable with those of the total group. Therefore, owing to the inclusion of 15–18-year-old adults in the general adult population in Denmark, a large group still enrolled in schools, the reported differences in the educational level are likely to be even more pronounced than what our results suggest.

### Implications

Our results add to the existing knowledge about people with physical impairments at a population level, as our population included all adults diagnosed with physical impairments at a private or public hospital in Denmark. Given that hospitalisation in Denmark is free, resulting in high levels of medical accessibility and correspondingly low levels of health disparity, the study population could closely resemble all adults in Denmark.

People with physical impairments are often treated as a homogeneous group, but our results illustrate that they should be regarded as a heterogeneous group. The subgroups differed significantly both in the physical impact of their impairment and in the socio-demographic and socio-economic characteristics, including the educational level, affiliation to the labour market, and thus income.

Socio-demographic and socio-economic factors should be considered when promoting recreational physical and social activities for people with impairments. These factors, mainly the educational level, income, and occupation, are known to influence the level of and possibilities of performing these activities among people without disabilities and may therefore also be relevant for people with physical impairments. A high socio-economic status is generally related to high recreational physical activity levels [42], and a high income increases the use of structured recreational physical activities, as memberships, for example, can be costly [43]. The marital status can indicate the level of social and physical support in relation to participation in social and physical activities.

## Conclusion

This study investigated adults with physical impairments in Denmark. We identified nine subgroups based on ICD-10 diagnoses (*n* = 606,857) from the DNPR. The total group represented 13% of the adult Danish population, and the most prevalent disorder was osteoarthritis, affecting 69% of all Danish people with physical impairments. We demonstrated significant differences in the socio-demographic and socio-economic profiles between the total group and the general Danish population as well as between the nine diagnostic subgroups. These findings must be considered when facilitating inclusion of people with physical impairments in societal activities.

## Supplementary Information

**Additional file 1.** Diagnostic subgroups by ICD-10 codes.

**Additional file 2.**

## Data Availability

The data that support the findings of this study are available from Statistics Denmark, but restrictions apply to the availability of these data, which were used under license for the current register-based study, and so are not publicly available. Data are however available from the authors upon reasonable request and with permission of Statistics Denmark.

## Abbreviations

DNPR: Danish National Patient Register
ICD-10: International Classification of Diseases 10th revision
ISCED: International Standard Classification of Education
WHO: World Health Organization

## Declarations

All data were obtained from already existing administrative national registers (no human participants were present) and accessed through the server at Statistics Denmark. The STROBE guidelines were used for study reporting. All methods were performed in accordance with relevant guidelines and regulations, including the Declaration of Helsinki.
