# Supplementary material for: Physical impairments among adults in Denmark: a register-based study
Source: BMC Public Health. 2022 Dec 23;22:2416. doi: 10.1186/s12889-022-14747-9 (PMC9783972; doi:10.1186/s12889-022-14747-9)
Supplement: Supplementary file 1 — Additional file 1. Diagnostic subgroups by ICD-10 codes. [file 12889_2022_14747_MOESM1_ESM.pdf]

Additional file 1

Diagnostic subgroups by ICD-10 codes.

Disability group	ICD-10 codes	Codename in the Danish SKS/ICD-10 system https://medinfo.dk/sks/brows.php	ICD-10 codes	English codes https://icd.who.int/browse10/2016/en - /I61.0
**Osteoarthritis**	DM16*	Slidgigt i hofte	M16*	Coxarthrosis [arthrosis of hip]
	DM17*	Slidgigt i knæ	M17*	Gonarthrosis [arthrosis of knee]
	DM19*	Andre former for slidgigt	M19*	Other arthrosis
**Acquired brain injury**	DI61*	Hjerneblødning	I61*	Intracerebral haemorrhage
	DI63*	Hjerneinfarkt	I63*	Cerebral infarction
	DI64*	Slagtilfælde uden oplysning om blødning eller infarkt	I64*	Stroke, not specified as haemorrhage or infarction
	DI691	Senfølge efter tidligere hjerneblødning	I69.1	Sequelae of intracerebral haemorrhage
	DI693	Senfølge efter tidligere hjerneinfarkt	I69.3	Sequelae of cerebral infarction
	DI694	Hjerneblødning	I69.4	Sequelae of stroke, not specified as haemorrhage or infarction
	DS020*	Fractura thecae cranii	S02.0*	Fracture of vault of skull
	DS021*	Fractura baseos cranii	S02.1*	Fracture of base of skull
	DS027*	Fractura multiplex cranii eet ossis faciei	S02.7*	Multiple fractures involving skull and facial bones
	DS028*	Kraniebrud og brud af ansigtets knogler, andre former	S02.8*	Fractures of other skull and facial bones
	DS029*	Kraniebrud og brud af ansigtets knogler uden specifikation	S02.9*	S02.9 Fracture of skull and facial bones, part unspecified
	DS061	Oedema cerebri traumaticum	S06.1	S06.1 Traumatic cerebral oedema
	DS062*	Laesio traumatica cerebri duffusa	S06.2*	S06.2 Diffuse brain injury
	DS063*	Laesio traumatica cerebri focalis	S06.3*	Focal brain injury
	DS064*	Haemorrhagia epiduralis traumatica	S06.4*	S06.4 Epidural haemorrhage
	DS065*	Haemorrhagia subduralis traumatica	S06.5*	S06.5 Traumatic subdural haemorrhage
	DS066	Haemorrhagia subarachnoidalis traumatica	S06.6	S06.6 Traumatic subarachnoid haemorrhage
	DS067	Laesio traumatica intracranialis m protraheret coma	S06.7	Intracranial injury with prolonged coma
	DS068*	Interkranielle læsioner, andre	S06.8*	S06.8 Other intracranial injuries
	DS069	Interkranielle læsioner uden specification	S06.9	Intracranial injury, unspecified
	DS070	Conquassatio faciei	S07.0	Crushing injury of face
	DS071	Conquassatio cranii	S07.1	S07.1 Crushing injury of skull
	DS079	Laesio traumatica multiplex capitis	S07.9	S07.9 Crushing injury of head, part unspecified
	DT020	Frakturer både på hoved og hals	T02.0	T02.0 Fractures involving head with neck
	DT040	Conquassatio både hoved og hals	T04.0	Crushing injuries involving head with neck
	DT060	Læsion af hjerne hjernenerver med spin el. nerver på hals	T06.0	T06.0 Injuries of brain and cranial nerves with injuries of nerves and spinal cord at neck level
**Rheumatoid arthritis**	DM05*	Seropositiv leddegigt	M05*	Seropositive rheumatoid arthritis
	DM06*	Andre former for leddegigt	M06*	Other rheumatoid arthritis
**Multiple sclerosis**	DG35*	Dissemineret sklerose	G35*	Multiple sclerosis

**Spinal cord injuries**	DG82*	Paraplegi og tetraplegi	G82*	Paraplegia and tetraplegia
	DM471C	Spondylose i halshvirvelsøjlen med myelopati og tetraplegi	M47.1	Other spondylosis with myelopathy<br>(Danish subcategory: 'Cervical spondylosis with myelopathy and tetraplegia')
	DT144C	Traumatisk paraplegi UNS	T14.4	Injury of nerve(s) of unspecified body region<br>(Danish subcategory: 'Traumatic paraplegia, unspecified')
	DT144D	Traumatisk tetraplegi UNS		
	DG114	Arvelig spastisk paraplegi	G11.4	Hereditary spastic paraplegia
	DQ05*	Spina bifida	Q05*	Spina bifida
	DQ760	Spina bifida occulta	Q76.0	Spina bifida occulta
**Cerebral palsy**	DG80*	Cerebral parese	G80*	Cerebral palsy
**Amputations**	DS48*	Traumatisk amputation af skulder og overarm	S48*	Traumatic amputation of shoulder and upper arm
	DS58*	Traumatisk amputation af albue og underarm	S58*	Traumatic amputation of forearm
	DS68	Traumatisk amputation af håndled og hånd	S68	Traumatic amputation of wrist and hand
	DS684	Traumatisk amputation af hånd	S68.4	Traumatic amputation of hand at wrist level
	DS688	Traumatisk amputation af anden del af håndled eller hånd	S68.8	Traumatic amputation of other parts of wrist and hand
	DS689	Traumatisk amputation af håndled eller hånd UNS	S68.9	Traumatic amputation of wrist and hand, level unspecified
	DS78*	Traumatisk amputation af hofte og lår	S78*	Traumatic amputation of hip and thigh
	DS88*	Traumatisk amputation i knæregion eller underben	S88*	Traumatic amputation of lower leg
	DS98	Traumatisk amputation af ankel og fod	S98	Traumatic amputation of ankle and foot
	DS983	Traumatisk amputation af anden del af fod	S98.3	Traumatic amputation of other parts of foot
	DS984	Traumatisk amputation af fod UNS	S98.4	Traumatic amputation of foot, level unspecified
	DT05*	Traumatisk amputation af flere legemsdele	T05*	Traumatic amputations involving multiple body regions
	DT116	Traumatisk amputation på arm UNS	T11.6	Traumatic amputation of upper limb, level unspecified
	DT136	Traumatisk amputation på ben UNS	T13.6	Traumatic amputation of lower limb, level unspecified
	DT926	Følgetilstand efter knusningslæsion eller traumatisk amputation på overekstremitet	T92.6	Sequelae of crushing injury and traumatic amputation of upper limb
	DT936	Følgetilstand efter knusningslæsion eller traumatisk amputation på underekstremitet	T93.6	Sequelae of crushing injury and traumatic amputation of lower limb
**Muscular dystrophy**	DG71*	Primære muskelsygdomme	G71*	Primary disorders of muscles
**Poliomyelitis**	DA80*	Akut polio	A80*	Acute poliomyelitis
	DB91	Følger efter polio	B91	Sequelae of poliomyelitis
	DG14	Postpoliosyndrom	G14	Postpolio syndrome

* indicates all sub codes are included
