# Supplementary material for: Physical impairments among adults in Denmark: a register-based study
Source: BMC Public Health. 2022 Dec 23;22:2416. doi: 10.1186/s12889-022-14747-9 (PMC9783972; doi:10.1186/s12889-022-14747-9)
Supplement: Supplementary file 2 — Additional file 2. [file 12889_2022_14747_MOESM2_ESM.pdf]

Additional file 2:

Pearson's chi-square test of independence was used to test for difference in distributions of the different variables for each of the eight impairment subgroups and the total impairment group, compared with the general adult population[2] in Denmark (each disability group is subtracted from the total group of adults in Denmark before comparison). Missing values and unknown values are excluded from the calculations. Test statistics ($\chi 2$) are presented with degrees of freedom, sample size, followed by the chi-square and p-values.

**a:** Sex (degree of freedom = 1)

| | sample size | chi-square value | p-value |
|---|---|---|---|
| Impairment subgroups: | | | |
| Osteoarthritis | 409,202 | 4.6e+03 | ≤0.001 |
| Acquired brain injury | 176,276 | 3.6e+03 | ≤0.001 |
| Rheumatoid arthritis | 40,590 | 7.4e+03 | ≤0.001 |
| Multiple sclerosis | 15,496 | 2.2e+03 | ≤0.001 |
| Spinal cord injuries | 8,922 | 242.2057 | ≤0.001 |
| Cerebral palsy | 7,336 | 109.8339 | ≤0.001 |
| Amputation | 4,191 | 925.9550 | ≤0.001 |
| Muscular dystrophy | 2,751 | 16.2064 | ≤0.001 |
| Total impairment group | 606,857 | 1.5e+03 | ≤0.001 |

**b:** Age male (degree of freedom = 6)

| | sample size | chi-square value | p-value |
|---|---|---|---|
| Impairment subgroups: | | | |
| Osteoarthritis | 181,420 | 1.9e+05 | ≤0.001 |
| Acquired brain injury | 99,439 | 9.7e+04 | ≤0.001 |
| Rheumatoid arthritis | 11,409 | 9.2e+03 | ≤0.001 |
| Multiple sclerosis | 4,742 | 1.3e+03 | ≤0.001 |
| Spinal cord injuries | 5,140 | 50.1350 | ≤0.001 |
| Cerebral palsy | 4,071 | 2.0e+03 | ≤0.001 |
| Amputation | 3,054 | 366.3647 | ≤0.001 |
| Muscular dystrophy | 1,464 | 47.5147 | ≤0.001 |
| Total impairment group | 285,391 | 2.5e+05 | ≤0.001 |

**c:** Age female (degree of freedom = 6)

| | sample size | chi-square value | p-value |
|---|---|---|---|
| Impairment subgroups: | | | |
| Osteoarthritis | 227,782 | 2.6e+05 | ≤0.001 |
| Acquired brain injury | 76,837 | 9.1e+04 | ≤0.001 |
| Rheumatoid arthritis | 29,181 | 1.6e+04 | ≤0.001 |
| Multiple sclerosis | 10,754 | 2.9e+03 | ≤0.001 |
| Spinal cord injuries | 3,782 | 6.7827 | 0.341 |

| | | | |
|---|---|---|---|
| Cerebral palsy | 3,265 | 1.3e+03 | ≤0.001 |
| Amputation | 1,137 | 90.6126 | ≤0.001 |
| Muscular dystrophy | 1,287 | 49.1435 | ≤0.001 |
| Total impairment group | 321,466 | 3.0e+05 | ≤0.001 |

**d:** Geographical region (degree of freedom = 4)

| | sample size | chi-square value | p-value |
|---|---|---|---|
| Impairment subgroups: | | | |
| Osteoarthritis | 409,202 | 5.4e+03 | ≤0.001 |
| Acquired brain injury | 176,276 | 1.5e+03 | ≤0.001 |
| Rheumatoid arthritis | 40,590 | 847.5063 | ≤0.001 |
| Multiple sclerosis | 15,496 | 64.0768 | ≤0.001 |
| Spinal cord injuries | 8,922 | 43.2551 | ≤0.001 |
| Cerebral palsy | 7,336 | 97.1720 | ≤0.001 |
| Amputation | 4,191 | 410.0054 | ≤0.001 |
| Muscular dystrophy | 2,751 | 4.2969 | 0.367 |
| Total impairment group | 606,857 | 6.2e+03 | ≤0.001 |

**e:** Origin (degree of freedom = 2)

| | sample size | chi-square value | p-value |
|---|---|---|---|
| Impairment subgroups: | | | |
| Osteoarthritis | 409,202 | 1.9e+04 | ≤0.001 |
| Acquired brain injury | 176,276 | 9.0e+03 | ≤0.001 |
| Rheumatoid arthritis | 40,590 | 1.6e+03 | ≤0.001 |
| Multiple sclerosis | 15,496 | 826.5158 | ≤0.001 |
| Spinal cord injuries | 8,922 | 212.9971 | ≤0.001 |
| Cerebral palsy | 7,336 | 558.2146 | ≤0.001 |
| Amputation | 4,191 | 57.6479 | ≤0.001 |
| Muscular dystrophy | 2,751 | 108.5948 | ≤0.001 |
| Total impairment group | 606,857 | 2.9e+04 | ≤0.001 |

**f:** Education level (degree of freedom = 3)

| | sample size | chi-square value | p-value |
|---|---|---|---|
| Impairment subgroups: | | | |
| Osteoarthritis | 401,684 | 2.9e+04 | ≤0.001 |
| Acquired brain injury | 172,321 | 1.9e+04 | ≤0.001 |
| Rheumatoid arthritis | 39,961 | 2.4e+03 | ≤0.001 |
| Multiple sclerosis | 15,368 | 126.0148 | ≤0.001 |
| Spinal cord injuries | 8,668 | 2.0e+03 | ≤0.001 |
| Cerebral palsy | 7,031 | 6.9e+03 | ≤0.001 |
| Amputation | 4,117 | 377.4903 | ≤0.001 |
| Muscular dystrophy | 2,716 | 167.0933 | ≤0.001 |

| | | | |
|---|---|---|---|
| Total impairment group | 595,259 | 4.7e+04 | ≤0.001 |

**g:** Occupation (degree of freedom = 4)

| | sample size | chi-square value | p-value |
|---|---|---|---|
| Impairment subgroups: | | | |
| Osteoarthritis | 405,608 | 3.6e+05 | ≤0.001 |
| Acquired brain injury | 174,185 | 1.8e+05 | ≤0.001 |
| Rheumatoid arthritis | 40,091 | 2.8e+04 | ≤0.001 |
| Multiple sclerosis | 15,272 | 2.1e+04 | ≤0.001 |
| Spinal cord injuries | 8,784 | 2.9e+04 | ≤0.001 |
| Cerebral palsy | 7,158 | 4.0e+04 | ≤0.001 |
| Amputation | 4,097 | 829.6559 | ≤0.001 |
| Muscular dystrophy | 2,698 | 5.1e+03 | ≤0.001 |
| Total impairment group | 600,314 | 5.1e+05 | ≤0.001 |

**h:** Marital status (degree of freedom = 3)

| | sample size | chi-square value | p-value |
|---|---|---|---|
| Impairment subgroups: | | | |
| Osteoarthritis | 409,202 | 1.8e+05 | ≤0.001 |
| Acquired brain injury | 176,276 | 6.4e+04 | ≤0.001 |
| Rheumatoid arthritis | 40,590 | 1.3e+04 | ≤0.001 |
| Multiple sclerosis | 15,496 | 1.1e+03 | ≤0.001 |
| Spinal cord injuries | 8,922 | 915.5575 | ≤0.001 |
| Cerebral palsy | 7,336 | 6.7e+03 | ≤0.001 |
| Amputation | 4,191 | 141.5728 | ≤0.001 |
| Muscular dystrophy | 2,751 | 193.0599 | ≤0.001 |
| Total impairment group | 606,857 | 2.1e+05 | ≤0.001 |
